# Feasibility of the Linus Health’s life and health questionnaire in primary‐care settings

**DOI:** 10.1002/alz.089695

**Published:** 2025-01-09

**Authors:** Marissa C Ciesla, Brendan Haas, Joyce Rios Gomes‐Osman, Russel Banks, Ali Jannati, John Showalter, Alvaro Pascual‐Leone

**Affiliations:** ^1^ Linus Health, Boston, MA USA; ^2^ Linus Health, Waltham, MA USA; ^3^ University of Miami Miller School of Medicine, Miami, FL USA; ^4^ Michigan State University, East Landing, MI USA; ^5^ Harvard Medical School, Boston, MA USA; ^6^ Hebrew SeniorLife, Boston, MA USA

## Abstract

**Introduction:**

Compelling evidence from longitudinal trials demonstrates that adopting certain lifestyles, especially in midlife to early late life, can reduce the risk of dementia, reduce the severity of the associated disability, and decrease progression to dementia in individuals with mild cognitive impairment by as much as 40%. The Life and Health Questionnaire (LHQ) is a 32‐item questionnaire – written at a 6th‐grade reading level – that captures information related to potentially modifiable lifestyle and psychosocial risk factors, and protective factors for cognitive decline and dementia. The objective of this study was to gain insights into the feasibility of the LHQ in primary care settings by evaluating the influence of cognition, age, and education on the LHQ completion times.

**Methods:**

We analyzed 1826 LHQ surveys between June and October 2023. Mann‐Whitney U tests were used to compare average LHQ completion times in relation to cognitive status as measured by the Digital Clock and Recall (DCR™), age, and education.

**Results:**

The average time to complete the LHQ among all patients was 4.8 minutes (median = 4.2 ± 2.4). Average LHQ completion times showed a gradual increase as DCR scores decreased (increasing cognitive impairment; mean = 3.5 and 6 minutes for DCR scores 5 and 0, respectively; p<0.05). There were no statistically significant differences in LHQ completion times as patient age increased. Those with higher education levels had significantly shorter average LHQ completion times (p<0.01).

**Conclusion:**

The LHQ is a brief survey capturing dementia risk and protective factors that takes an average of 4.8 minutes to complete. Completion time is lengthened with lower education levels and greater cognitive impairment to an average of 6 min. These results indicate the feasibility of the LHQ to be performed in primary‐care settings given its brief completion time.

